# Impact of Different Microbial Biostimulants and Salt Stress on the Endophytome of the Edible Part of Lettuce and Tomato Plants

**DOI:** 10.3390/foods14193366

**Published:** 2025-09-29

**Authors:** José M. Mulet, Patricia Benito, Marina Celdrán, Lynne Yenush, Rosa Porcel

**Affiliations:** 1 Instituto de Biología Molecular y Celular de Plantas (IBMCP), Universitat Politècnica de València-Consejo Superior de Investigaciones Científicas, 46022 Valencia, Spain; jmmulet@ibmcp.upv.es (J.M.M.); p.benito@caldic.com (P.B.); mcelalc@ibmcp.upv.es (M.C.); lynne@ibmcp.upv.es (L.Y.); 2 Caldic Ibérica, S. L. U. Llobateras 23-25, Pol. Ind. Santiga, Barberà del Vallés, 08210 Barcelona, Spain

**Keywords:** biostimulant, salt stress, *Priestia megaterium*, arbuscular mycorrhiza fungi, endophyte, lettuce, tomato

## Abstract

The human gut microbiota plays a critical role in health throughout life. While fruits and vegetables are well-known sources of nutrients and prebiotics, recent studies suggest they may also contribute viable microorganisms to the gut microbiome, particularly when consumed raw. However, the impact of agricultural practices—such as the use of microbial biostimulants or exposure to salt stress—on the composition of the edible plant microbiome remains poorly understood. In this study, we performed a comprehensive metataxonomic analysis of the endophytic microbiome in the edible tissues (leaves or fruits) of lettuce (*Lactuca sativa*) and tomato (*Solanum lycopersicum*), cultivated under standard conditions with or without microbial biostimulants and salt stress. Our results show that microbial biostimulants—*Priestia megaterium* (PGPB) and *Rhizophagus irregularis* (AMF)—as well as moderate salt stress, significantly reshape the composition and diversity of endophytes in both crops. Notably, the PGPB and NaCl treatments enhanced the abundance of bacterial genera such as *Pantoea*, *Stenotrophomonas*, and *Massilia*, which are associated with plant health and may have probiotic potential. Salt stress also increased alpha-diversity indices and favored the presence of *Firmicutes* and *Bacteroidota*, phyla commonly linked to a healthy human gut microbiome. Agronomic inputs used in organic and conventional farming, such as microbial biostimulants or controlled salt exposure, may represent novel strategies to enhance the microbial quality of fresh produce and promote gut microbial diversity through diet.

## 1. Introduction

Growing evidence shows an important interplay between diet and gut microbiome, with significant implications for health and disease [1]. Diet significantly influences gut microbiota composition [1,2]. Fruits and vegetables contain vitamins, nutrients, and bioactive plant secondary metabolites that can affect the diversity in the composition of the gut microbiome [3]. For instance, high fiber content is considered prebiotic for the gut microbiome, and diets high in fruit/legume fiber are associated with greater microbial diversity [4]. It has been hypothesized that the effect of fruit and vegetables on the diet would not only be prebiotic, but may also contribute as a source of environmental microbiota, which could serve as a genetic reserve for diversity and could be functional in the human host [5]. These questions are not easy to address, as the availability of large-scale food microbiome metagenomes is still minimal. Therefore, the view that fruit and vegetable-associated bacteria are underrepresented in human gut microbiomes may not represent the whole picture. Although it is true that the microbiota associated with crops such as cereals, pulses, or tubers, which are usually cooked, are likely to have a minimal impact on the human microbiota, many fruits and vegetables are eaten raw, so the bacteria from these plants may indeed colonize the gut.

In plants, there are different populations of microorganisms. The phyllosphere is the population of microorganisms inhabiting the above-ground surface of a plant, whereas the rhizosphere is the equivalent below ground. The phyllosphere and rhizosphere may suffer many alterations during cultivation and harvest. Many post-harvest treatments that prolong vegetable shelf life are based on removing viable microorganisms from the surface. It must also be considered that bleach-cleaning of fruits and vegetables, common in many parts of the world as an elementary sanitary practice, may also eliminate the phyllosphere associated with many vegetables. It is clear that all of these sanitary treatments are focused on the surface of the vegetables. Interestingly, there is a third well-defined population of microorganisms in a plant, the endophytome. This microbiome comprises microorganisms that colonize the inside of a given host plant [6]. The post-harvest treatments cited above are likely to have a minimal impact on the internal endophytome of fruits. Therefore, if there is a source of vegetable-associated microbes that could impact the gut microbiome, it is more likely to come from the endophytome of the edible part of fruits and vegetables, which are commonly consumed raw.

In recent years, the use of biostimulants has gained interest. Biostimulants are, according to du Jardin, 2015, “a substance or microorganism that, when applied to seeds, plants, or on the rhizosphere, stimulates natural processes to enhance or benefit nutrient uptake, nutrient use efficiency, tolerance to abiotic stress, or crop quality and yield” [7]. Non-microbial biostimulants and microbial biostimulants, such as bacteria (mainly plant growth-promoting bacteria (PGPB)) or fungi (mostly arbuscular mycorrhizal fungi (AMF)), are widely used in organic farming as they are of natural origin. They are also gaining popularity in conventional agriculture due to the increasing restrictions on the use of fertilizers and pesticides in most agricultural regulations. There is a significant gap in our knowledge of how different cultivation methods or agrarian inputs, such as the above-mentioned microbial biostimulants, may affect the plant endophytome. It is commonly considered that organic farming practices enrich the microbiome associated with the crops, as they use natural fertilizers. It is also believed that industrial farming practices, such as hydroponic cultivation, due to the confined environment or the use of antibiotics and pesticides, may lead to plants with fewer microorganisms [8]. However, few studies have been conducted characterizing the endophytome of fruits and vegetables cultivated using different agricultural inputs, farming practices, or in the presence of microbial biostimulants.

There is also another factor that must be considered in addition to farming practices. Anthropogenic global warming significantly affects natural environments [9] and agricultural production [10]. Most horticultural production areas face extended dry periods and increased temperatures and aridity. A secondary effect of these changes in the rain patterns is the increase in soil salinization due to high rates of extraction from aquifers, which facilitates the intrusion of seawater, and the fact that extensive irrigation induces salt accumulation in the soil. Among all the environmental constraints that horticultural production must face, salt stress is more likely to affect the endophytome. This is because potassium is the central cation in the plant’s internal media. Plant cells energize their membrane using a potassium gradient, contrary to animal cells, which use a sodium gradient. In addition, potassium has many functions in plant biochemistry and physiology, such as being the primary solute to maintain cell turgor and creating the appropriate ionic environment for enzymatic function, among others [11]. Under salt stress, sodium enters the plant, creating ionic toxicity and altering the potassium concentration [11]. Moreover, in many cases, sodium is taken up by the roots, loaded into the xylem, transported to the aerial part, and accumulated in vacuoles. That means that upon salt stress, there is an alteration of the ion homeostasis of the internal vascular system of the plants, where most of the endophytome microbes are located. Fungi and some bacteria also use potassium to energize the membrane in a similar way to plants [12], so a change in the internal ionic environment may be detrimental for many microorganisms, therefore altering the equilibrium, mainly affecting the salt-sensitive phylum and creating an environment where the proliferation of salt-tolerant species may be favored. It is known that some agricultural inputs can counteract this effect, for instance, the arbuscular mycorrhizal (AM) symbiosis favors Na^+^ extrusion from the cytoplasm, its sequestration into the vacuole, the unloading of Na^+^ from the xylem, and its recirculation from photosynthetic organs to roots. In rice, for example, there is a decrease in the Na^+^ root-to-shoot distribution and an increase in Na^+^ accumulation in roots, which seems to enhance the plant’s tolerance to salinity and allows mycorrhizal rice plants to maintain their developmental program under salt conditions [13].

With this in mind, we hypothesized that salt stress during cultivation, a likely circumstance in many production areas like the Mediterranean basin, would significantly affect the equilibrium of the internal plant microbiome.

## 2. Materials and Methods

### 2.1. Soil and Biological Material

Lettuce seeds (*Lactuca sativa* L. cv. Romana) were superficially sterilized with commercial bleach diluted 1:1 (*v*/*v*) for 15 min and rinsed with sterile water until the disinfecting agent was removed. The germination of the seeds was carried out in seedbeds with coconut fiber in CUCALA AGRICOLA S.L. (38°56′42″ N, 0°25′42″ W. The assay was conducted during the autumn of 2022 (from 20 September 2022, to 9 November 2022). After one month, the seedlings were transplanted into 5 L pots and placed in a greenhouse at a distance of 15 cm between pots and in a zigzag arrangement to prevent inhibition of growth by competition and facilitate watering.

Seeds of tomato (*Solanum lycopersicum* cv. Micro-Tom) were disinfected by immersion in a 1:1 (*v*/*v*) solution of commercial bleach (Conejo, Helkel Ibérica, Montornés de Vallès, Barcelona) for 15 min, followed by thorough rinsing with sterile water. Germination took place on seedbeds filled with coconut fiber at CUCALA AGRÍCOLA S.L. facilities in Beniganim, Spain (38°56′42″ N, 0°25′42″ W). The initial phase of the experiment was conducted between 15 May and 9 June 2022. After approximately four weeks, the seedlings were transplanted into 5 L pots, arranged in a greenhouse with 15 cm spacing between containers. The greenhouse trial with tomato plants was performed during the summer season of 2022, spanning from 9 June to 16 August 2022.

The substrate employed for plant growth was prepared by combining pasteurized Terra Vagiota substrate (Valagussa Group, Merate, Italy) with river sand. For lettuce cultivation, the mixture was formulated at a 1:1 ratio (*v*/*v*), whereas for tomato cultivation, the proportion was adjusted to 2:1 (*v*/*v*).

### 2.2. Inoculation Treatments

The non-microbial biostimulant Calbio (Caldic Ibérica S.L.U, Barcelona, Spain) was used at a concentration of 200 μg/mL. In previous research, this biostimulant was formulated from a combination of four natural extracts in our laboratory [14,15]. Specifically, 10 mL of Calbio per plant was applied 2 and 15 days (tomato experiment) or 4 and 15 days (lettuce experiment) after transplantation. The AMF used was *Rhizophagus irregularis* strain RI6E6, a commercial fungal strain from Microbiol Laboratories SLU (Tarragona, Spain; catalog number CAEP5M14FN-I). Furthermore, 5 g of inoculum (600 infectious propagules per gram of product) was added at the time of transplantation per pot. On the other hand, the PGPB strain used in these assays was *Priestia megaterium* BM08, provided by the company Ceres Biotic Tech S.L (San Fernando de Henares, Spain; catalog number CB24). At one week after transplantation, 5 mL of bacterial suspension (1.8 × 109 CFU/mL) was inoculated at the base of the stem. The dose of Calbio was previously determined by Benito et al., 2024; Benito et al., 2025 [16,17] while the inoculum of microorganisms was established from previous analyses of its ability to colonize the rhizosphere and promote growth in white clover (*Trifolium repens*) plants.

Control plants without mycorrhizal treatment were supplied with an equivalent quantity of autoclaved mycorrhizal inoculum. In addition, each plant received a 10 mL aliquot of a filtrate (<20 μm) derived from the AMF inoculum, ensuring the presence of a general soil microbial community while excluding AMF propagules. Similarly, non-inoculated control plants received the same number of applications with the same growth medium without bacteria.

### 2.3. Experimental Design and Growth Conditions

The field experiments were designed based on traditional tomato and lettuce cultivation practices in the Mediterranean basin. The experimental field was covered with high-density polypropylene mesh to prevent excessive heat and deter birds, following standard cultivation procedures for planting summer tomatoes and fall lettuce.

#### 2.3.1. Lettuce Experiment

The experiment consisted of a randomized complete block design with five treatments: (1) control plants grown without any biostimulants (Control); (2) plants treated with a combination of a non-microbial-based biostimulant, Calbio, and the microbial-based biostimulant BM08 (Calbio + PGPB); (3) plants treated with a microbial-based biostimulant containing the arbuscular mycorrhizal fungus *Rhizophagus irregularis* strain RI6E6 (AMF); (4) plants treated only with the microbial-based biostimulant BM08 (PGPB); and (5) plants grown under saline conditions (NaCl). Each treatment had 20 replicates, giving a total of 100 pots.

The trial was conducted during the autumn of 2022 (from 20 September 2022 to 9 November 2022) at the Centro de Experiencias Cajamar (Paiporta, Valencia) (39°25′01″ N, 0°25′04″ W).

Irrigation was carried out with nutrient solution (13 mM NO_3_^−^, 0.3 mM H_2_PO_4_^−^, 3.24 mM SO_4_^2−^, 1 mM HCO_3_^−^, 4.8 mM Cl^−^, 1.6 mM NH_4_^+^, 6.1 mM K^+^, 4.6 mM Ca^2+^, 2.8 mM Mg^2+^ and 2.3 mM Na^+^, EC = 2.5 mS/cm and pH 6) supplied to each pot by daily drip, except for the days when salt stress was applied. Salt stress was applied 4 weeks after transplantation, and the process was intensified in daily increments of approximately 40 mM NaCl to avoid osmotic shock to a final concentration of 100 mM. Subsequently, each pot was watered with 100 mL of 100 mM NaCl solution for 22 days until the crop was fully harvested. Plants in which no stress was applied were irrigated with 100 mL of distilled water or nutrient solution without added NaCl.

The harvest was carried out when the lettuce heads were formed entirely on 9 November 2022. After harvesting, leaf samples were rinsed and frozen in liquid nitrogen and stored at −80 °C until analysis.

#### 2.3.2. Tomato Experiment

The experiment consisted of a factorial design with five treatments: (1) control plants grown without any biostimulants (Control); (2) plants treated with a combination of a non-microbial-based biostimulant, Calbio, and the microbial-based biostimulant BM08 (Calbio + PGPB); (3) plants treated with a microbial-based biostimulant containing the arbuscular mycorrhizal fungus RI6E6 (AMF); (4) plants treated only with the microbial-based biostimulant BM08 (PGPB), and (5) plants subjected to salt stress (NaCl). Each treatment had 20 replicates, totaling 100 pots.

The trial was carried out during the summer of 2022 (from 9 June 2022, to 16 August 2022) at the Centro de Experiencias Cajamar (Paiporta, Valencia) (39°25′01″ N, 0°25′04″ W).

Irrigation was performed with nutrient solution (13 mM NO_3_^−^, 0.3 mM H_2_PO_4_^−^, 3.24 mM SO_4_^−2^, 1 mM HCO_3_^−^, 4.8 mM Cl^−^, 1.6 mM NH_4_^+^, 6.1 mM K^+^, 4.6 mM Ca^+2^, 2.8 mM Mg^+2^ and 2.3 mM Na^+^, EC = 2.5 mS/cm and pH 6) supplied to each pot by daily drip (except the days that saline stress was applied). In the case of plants subjected to salinity, stress was applied 4 weeks after planting; the process was stepped up in roughly 20 mM NaCl daily increments to avoid osmotic shock until reaching a final concentration of 100 mM. Thereafter, each pot was irrigated with 70 mL of 100 mM NaCl solution for 46 days until the crop was harvested entirely. Where no stress was applied, plants were irrigated with 70 mL of distilled water or nutrient solution without added NaCl.

After harvesting, leaf samples were rinsed and frozen in liquid nitrogen and stored at −80 °C until analysis.

### 2.4. Bacterial Colonization of the Rhizosphere and Symbiotic Development

Bacterial colonization by the plant growth-promoting bacterium (*Pseudomonas megaterium* strain BM08) was assessed through isolation from rhizosphere samples. Under aseptic conditions, rhizosphere material was weighed and suspended in 100 mL of sterile distilled water, followed by incubation for 15 min at room temperature. Serial dilutions were subsequently prepared, and aliquots from the 10^−3^ and 10^−4^ dilutions were plated onto yeast extract peptone dextrose (YPD) medium (1% yeast extract, 2% bacteriological peptone, 2% glucose, and 2% agar in distilled water) [18], as well as onto tryptone soy agar (TSA; 3% trypticase soy broth and 2% agar in distilled water). Plates were incubated at 28 °C for 4 days, after which colony-forming units (CFU/mL) were quantified. Colonies displaying morphological characteristics consistent with strain BM08 were subsequently isolated, and their identity was confirmed by amplification and sequencing of the 16S rRNA gene using primers previously described in [19].

The percentage of mycorrhizal root infection was estimated by visual observation of fungal colonization after clearing washed roots in 10% KOH, acidified with 1% HCl, and staining with 0.05% trypan blue in lactic acid (*v*/*v*), according to [20]. The extent of mycorrhizal colonization was calculated according to the gridline intersect [21].

### 2.5. Microbial Metagenomic DNA Extraction and Quantification

To avoid interference in the results, previous washes were carried out on tomatoes and lettuce leaves to eliminate the phyllosphere. Total DNA was extracted using the Dneasy Plant Mini kit (Qiagen, Venlo, The Netherlands) as it is efficient for endophytes [22]. Total DNA was quantified using Qubit dsDNA High Sensitivity technology (Invitrogen, Carlsbad, CA, USA).

### 2.6. High Throughput Sequencing of 16S rRNA and ITS2 Genes and Bioinformatics Analysis

The libraries were prepared according to the manufacturer’s protocol (Illumina, San Diego, CA, USA). The 341F (5′-CCTAYGGGRBGCASCAG-3′) and 806R (5′-GGACTACNNGGGTATCTAAT-3′) universal primers were used to amplify the conserved V3 and V4 regions of the 16S rRNA gene. DNA amplicon libraries from the ITS2 region were generated using the ITS3-F_KYO2 (5′-GATGAAGAACGYAGYRAA-3′) and ITS4_KYO1 (5′-TCCTCCGCTTWTTGWTWGC-3′ primers. The amplicons were sequenced using the Illumina HiSeq platform (2 × 300 bp). The full description of the library amplification and preparation protocol can be found in Safari et al., 2020 [23].

The raw sequences generated by Illumina were imported into the bioinformatics tool Qiime2 [24] to carry out an initial quality control process of the sequences with DADA2. The taxonomic mapping of each amplicon sequence variant (ASV), defined at a sequence similarity of 99.9%, was performed using the classify-Sklearn module in combination with the SILVA v138 [25] and UNITE (v. 9.0) databases for the taxonomic mapping of 16S rRNA and ITS2, respectively. Statistical and microbial ecology analyses were performed using different R software packages (v4.3.1; R Core Team 2023), including Phyloseq [26] and Vegan [27]. Significant differences in the relative abundances of the genus were performed using the MaAsLin2 R package (v. 1.0.0) [28] with the following parameters: min_abundance = 0, min_prevalence = 0.05, max_significance = 0.05, normalization = ‘None,’ transform = ‘LOG,’ analysis_method = “LM,” correction = “BH,” standardize = FALSE.

The number of readings ranged between 20,000 and 200,000 reads. Samples with few reads (fewer than 1000) were discarded for not presenting sufficient sequencing depth. Samples were filtered following the standard protocols for this kind of analysis to discard mitochondrial and chloroplast sequences. The samples’ rarefaction curves were saturated, indicating that the sequencing depth was adequate to detect all the diversity in the samples (Supplementary Figures S1 and S2). The number of sequences and the filtering results are provided in Supplementary Table S1.

## 3. Results

The objective of the present study was to investigate whether standard agricultural inputs, such as microbial biostimulants or the presence of salt stress, could have an impact on the composition of the endophytome of the edible part (leaf or fruit) of two horticultural crops, which are usually eaten raw (lettuce and tomato). We planned an experimental design for soil cultivation without any treatment, adding a PGPB (*Priestia megaterium* strain BM08) and an AM fungus (*Rhizophagus irregularis* strain RI6E6). These symbiotic microorganisms are present in the rhizosphere and are widely used in organic and conventional farming. We also tested the effect of NaCl, as this is a common constraint in many areas of tomato and lettuce cultivation. Additionally, we tested the PGPB in combination with the non-microbial biostimulant Calbio. The rationale behind this is that we have shown in two recent reports that combining this biostimulant with PGPB under standard growth conditions is more effective in increasing yield than treatment with Calbio alone [16,17]. Current research in our lab suggests that Calbio could exert its effect on the PGPB, rather than directly on the plant. Therefore, we did not include the Calbio treatment in this study, as the effects are quite minor and pre-experiments indicate little difference with respect to the control. A complete description of the effect of these treatments on plant physiology and biochemistry, and the basic compositional traits can be found in [16,17].

### 3.1. Bacterial Communities

**Figure 1:**
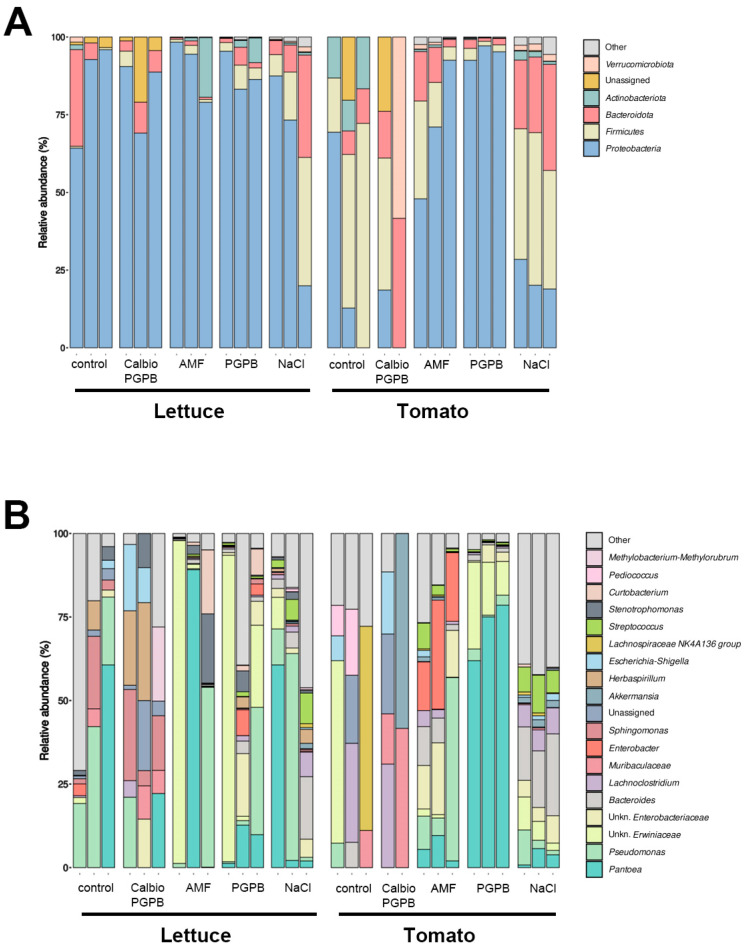
Relative abundance of phyla and genus present in endophytomes associated with the edible part of lettuce and tomato under different treatments. (**A**) Taxonomic distribution at the phylum level of the bacteria present in three biological replicates for each condition is indicated. (**B**) Taxonomic distribution at the genus level of the bacteria present in three biological replicates for each condition is stated. The treatments are control plants grown without any biostimulants (Control); plants treated with a combination of a non-microbial-based biostimulant, Calbio, and the microbial-based biostimulant BM08 (Calbio + PGPB); plants treated with a microbial-based biostimulant containing the arbuscular mycorrhizal fungi RI6E6 (AMF); and plants with the salt treatment (NaCl). The analyzed organs are lettuce leaf (Lettuce) or tomato fruit (Tomato).

At the phylum level, it was seen that in most of the lettuce samples, Proteobacteria (Pseudomonadota) was predominant, as described in previous studies [29], being the dominant phylum in all of the treatments. However, the treatments, especially NaCl, affected the relative abundance (Figure 1A). The phylum Bacteroidota was also an important component in the control group. However, it was only observed in greater abundance in the NaCl group compared to the AMF group. The phylum Firmicutes (Figure 1A) was abundant in the NaCl samples and significantly more present in these samples than in the AMF and PGPB groups. In these last two groups, the presence of Actinobacteriota was also noteworthy.

The tomato samples presented a very different composition and response to the treatments. Pseudomonadota, Bacillota, Bacteroidota, and Actinomycetota were the predominant phyla (Figure 1A). These results are in agreement with previous studies of the composition of tomato endophytes [30]. Compared to lettuce, noteworthy alterations in the relative abundance were observed in the treated samples. Specifically, Proteobacteria were abundant in the AMF treatment, especially in the PGPB samples. The PGPB species used in this study is BM08, belonging to the phylum Pseudomonadota [31], so the pronounced increase in the presence of this phylum in the PGPB group may be a consequence of the use of this bacterial species to promote plant growth. However, the phyla Firmicutes and Bacteroidota showed higher relative abundance in tomato than in lettuce, specifically under salt stress. Previous studies have described the presence of these two phyla in the endophytome of date palm under saline water irrigation conditions [32]. The untreated control had a higher relative abundance of Actinomycetota than the treated samples.

At the genus level, there were also significant differences between the two crops (Figure 1B). In the lettuce samples, the biological replicates tended not to be homogeneous within each group, making it difficult to conclude when comparing them. However, in the tomato samples, a greater correlation was observed between the different replicates within the same treatment, especially in AMF, PGPB, and NaCl. Especially interesting is the high relative abundance of the Pantoea genus in tomato treated with PGPB. The Pantoea genus has been described in field-grown tomatoes and can be used as an effective biocontrol agent against some fungal diseases [33].

### 3.2. Alpha-Diversity

We studied the alpha-diversity as a standard measure of sample diversity. Three alpha-diversity indices were calculated: observed richness, Shannon, and Simpson diversity. Observed richness represents the number of different amplicon sequence variants (ASVs), which indicates the presence of unique strains observed in each sample. The Shannon index is a measure of diversity that considers the number of different taxa and their relative abundances. The Simpson Index quantifies how sequences are distributed in each identified ASV, so a value of 1 indicates that all taxa have the same abundance [34].

**Figure 2:**
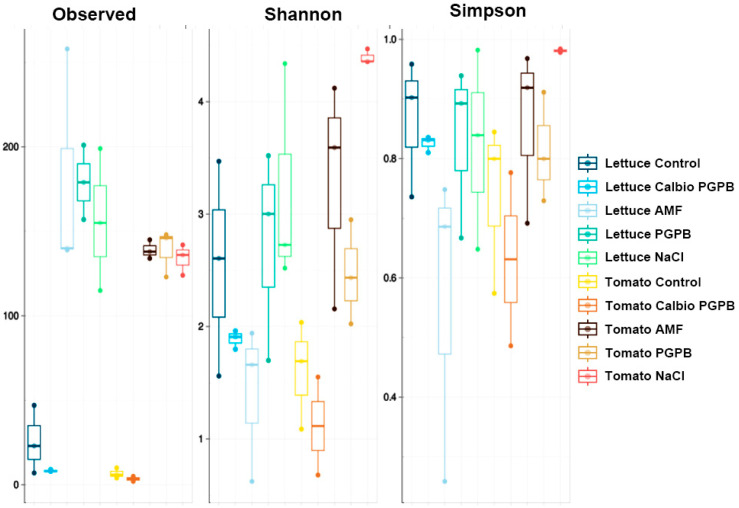
Results of alpha-diversity at the amplicon sequence variant (ASV) level were determined as the observed difference (observed), the Shannon index (Shannon), and the Simpson index (Simpson). The analyzed organs are lettuce leaf (Lettuce) or tomato fruit (Tomato). The treatments are control plants grown without any biostimulants (Control); plants treated with a combination of a non-microbial-based biostimulant, Calbio, and the microbial-based biostimulant BM08 (Calbio + PGPB); plants treated with a microbial-based biostimulant containing the arbuscular mycorrhizal fungi RI6E6 (AMF); and plants with the salt treatment (NaCl). *n* = 3.

Generally, a greater diversity was observed in lettuce than in tomato (Figure 2), although not in all treatments. The main differences observed in this parameter were found between the control and the AMF and PGPB groups in lettuce leaves and tomato fruits. However, these differences were not observed at the level of the Shannon and Simpson indices. The NaCl group only seemed to differ from the Calbio + PGPB group in the number of ASVs observed. In addition, this group showed Shannon and Simpson index values higher than the Calbio + PGPB and AMF groups in both crops. The latter group exhibits lower alpha-diversity values when comparing the control treatment with the Calbio + PGPB treatment. It is common to find a few endophytic bacterial species in both lettuce and tomato [29,30,35]. In this case, a low number of ASVs was detected in the control group and the BS + PGPB, although in the AMF, PGPB, and NaCl groups, between 100 and 200 more ASVs were found in each sample, figures similar to those seen in tomato [36] and even higher than those seen in similar studies in lettuce [37] (Figure 2). Therefore, the treatments significantly affected the richness of the samples. The most dramatic effect was observed in the Shannon and Simpson indices of NaCl-treated tomatoes, indicating that this treatment promotes biodiversity and normalizes the relative abundance of different taxa.

### 3.3. Beta-Diversity

**Figure 3:**
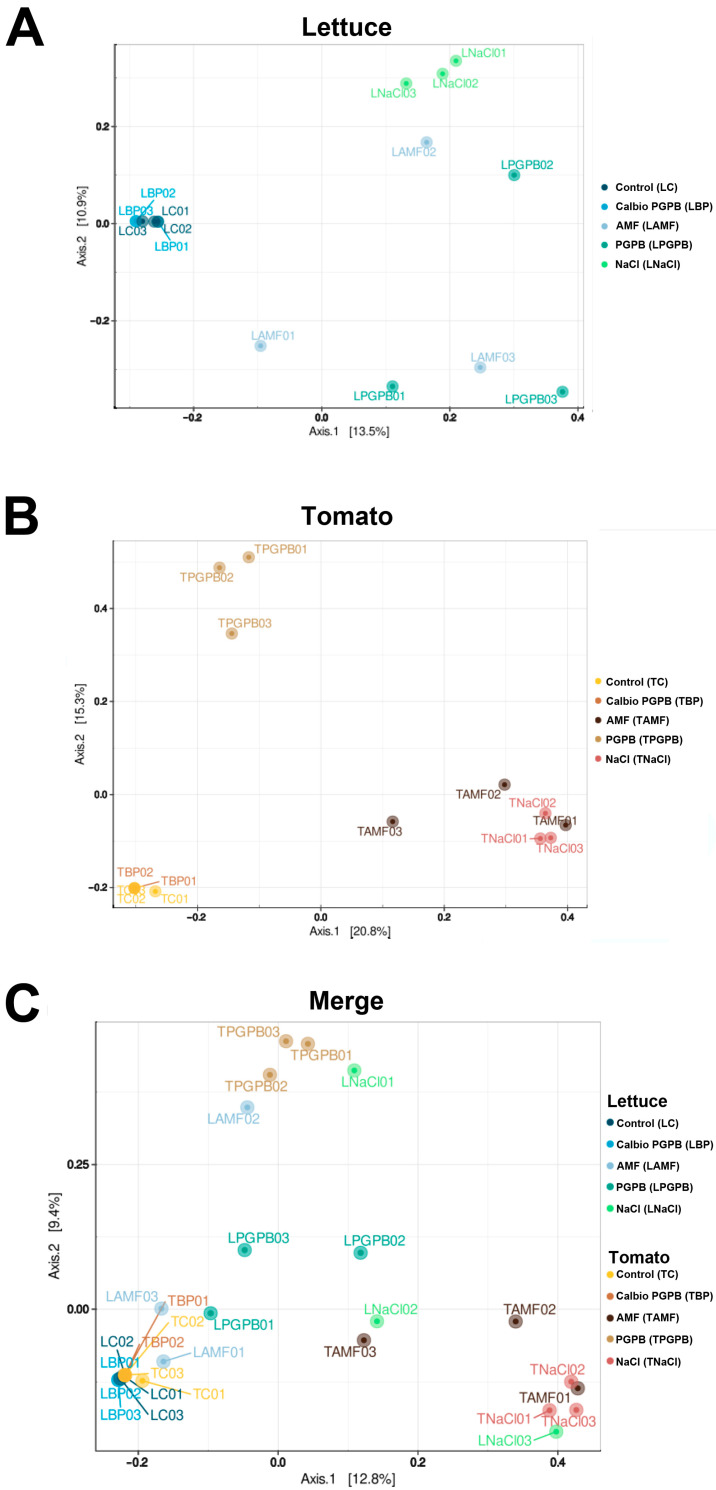
ASV-level β-diversity results of (**A**) lettuce samples, (**B**) tomato samples, and (**C**) both species merged. The treatments are control plants grown without any biostimulants (Control); plants treated with a combination of a non-microbial-based biostimulant, Calbio, and the microbial-based biostimulant BM08 (Calbio + PGPB); plants treated with a microbial-based biostimulant containing the arbuscular mycorrhizal fungi RI6E6 (AMF); and plants with the salt treatment (NaCl). Lettuce: Control (LC), Calbio + PGPB (LBP), AMF (LAMF), PGPB (LPGPB), and NaCl (LNaCl). Tomato: Control (TC), Calbio + PGPB (TBP), AMF (TAMF), PGPB (TPGPB), and NaCl (TNaCl). Different biological replicates are numbered 01, 02, and 03.

Beta-diversity refers to the differences in microbial community composition between samples or environments in the context of microbiomes. The beta-diversity study used principal component analysis (PCA) after calculating the distances between samples using the Bray–Curtis method [38]. The closer two points (=microbiomes) are located to each other in the two-dimensional space of the graph, the more similar they are in quantitative and qualitative terms. Most of the replicates that were part of the same group were generally close to each other, indicating the reproducibility of the biological replicates, the influence of the type of treatment on the microbial profiles, and consequently, the validity of our experimental design. In lettuce, the Calbio + PGPB and the control treatment were grouped. However, we found more diversity and differences among replicates in the other treatments, and no group was similar to the NaCl group (Figure 3A). Also, in tomato, the control and the Calbio + PGPB were grouped; PGPB was located in another part of the diagram, and AMF and NaCl presented similar results (Figure 3B). When we merged both results, it became clear that the controls without treatment were grouped, regardless of the plant species or organ analyzed (Figure 3C).

### 3.4. Fungal Communities

**Figure 4:**
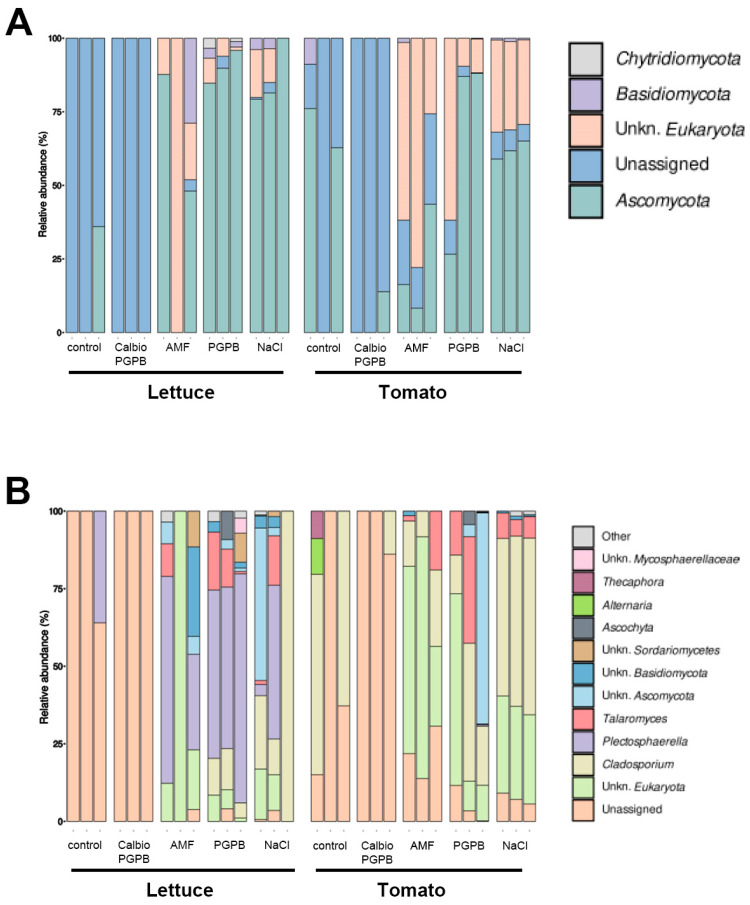
Relative abundance of phyla and genus present in endophytomes associated with the edible part of lettuce and tomato under different treatments. (**A**) Taxonomic distribution at the phylum level of the fungi present in the samples in three biological replicates for each condition is indicated. (**B**) Taxonomic distribution at the genus level of the fungi present in three biological replicates for each condition is stated. The treatments are control plants grown without any biostimulants (Control); plants treated with a combination of a non-microbial-based biostimulant, Calbio, and the microbial-based biostimulant BM08 (Calbio + PGPB); plants treated with a microbial-based biostimulant containing the arbuscular mycorrhizal fungi RI6E6 (AMF), and plants with the salt treatment (NaCl). The analyzed organs are lettuce leaf (Lettuce) or tomato fruit (Tomato).

We also investigated the effect of the different treatments on the endophytic fungi. In general, after filtering, the number of sequences from fungal origin was very low, indicating that the presence of fungi in the endophytome is limited. When analyzing the composition at the phylum level, a non-assigned phylum was the most prevalent in the lettuce control and Calbio + PGPB samples. Ascomycota was the most represented phylum in the PGPB and NaCl group, both in tomato and lettuce. An unknown phylum of the kingdom Eukaryota was represented in the AMF group (Figure 4A).

The genus *Plectosphaerella* predominated in lettuce, mainly in the PGPB group, although it was also seen in the AMF and NaCl groups. This genus was detected systematically in most of the lettuce samples, so it could be a standard endophyte of this crop, as seen in other plants [39]. In addition, the genus *Talaromyces* was also found in samples from the AMF, PGPB, and NaCl-treated groups, both in lettuce and tomato. The potential endophytic nature of this genus has already been described in recent studies [40]. *Cladosporium* was another genus in different treatments in both groups, predominant in the tomato control and NaCl groups. This genus is known for its endophytic role in tomatoes and lettuce and is also present in saline environments [41] (Figure 4B).

### 3.5. Alpha-Diversity

**Figure 5:**
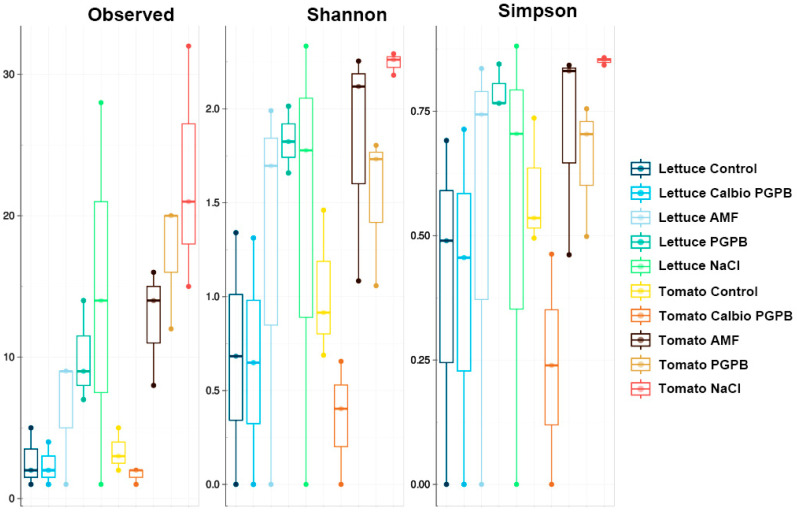
Results of α-diversity at the amplicon sequences variant (ASV) level are determined as the observed difference (observed), the Shannon index (Shannon), and the Simpson index (Simpson). The analyzed organs are lettuce leaf (Lettuce) or tomato fruit (Tomato). The treatments are control plants grown without any biostimulants (Control); plants treated with a combination of a non-microbial-based biostimulant, Calbio, and the microbial-based biostimulant BM08 (Calbio + PGPB); plants treated with a microbial-based biostimulant containing the arbuscular mycorrhizal fungi RI6E6 (AMF); and plants with the salt treatment (NaCl). *n* = 3.

We studied the alpha-diversity of the fungal communities in both lettuce and tomato. The treatment with AMF resulted in fewer observed ASVs. As observed in the bacterial communities, the samples with NaCl tended to show higher Shannon and Simpson indices than the rest of the groups, especially in tomato (Figure 5).

### 3.6. Beta-Diversity

**Figure 6:**
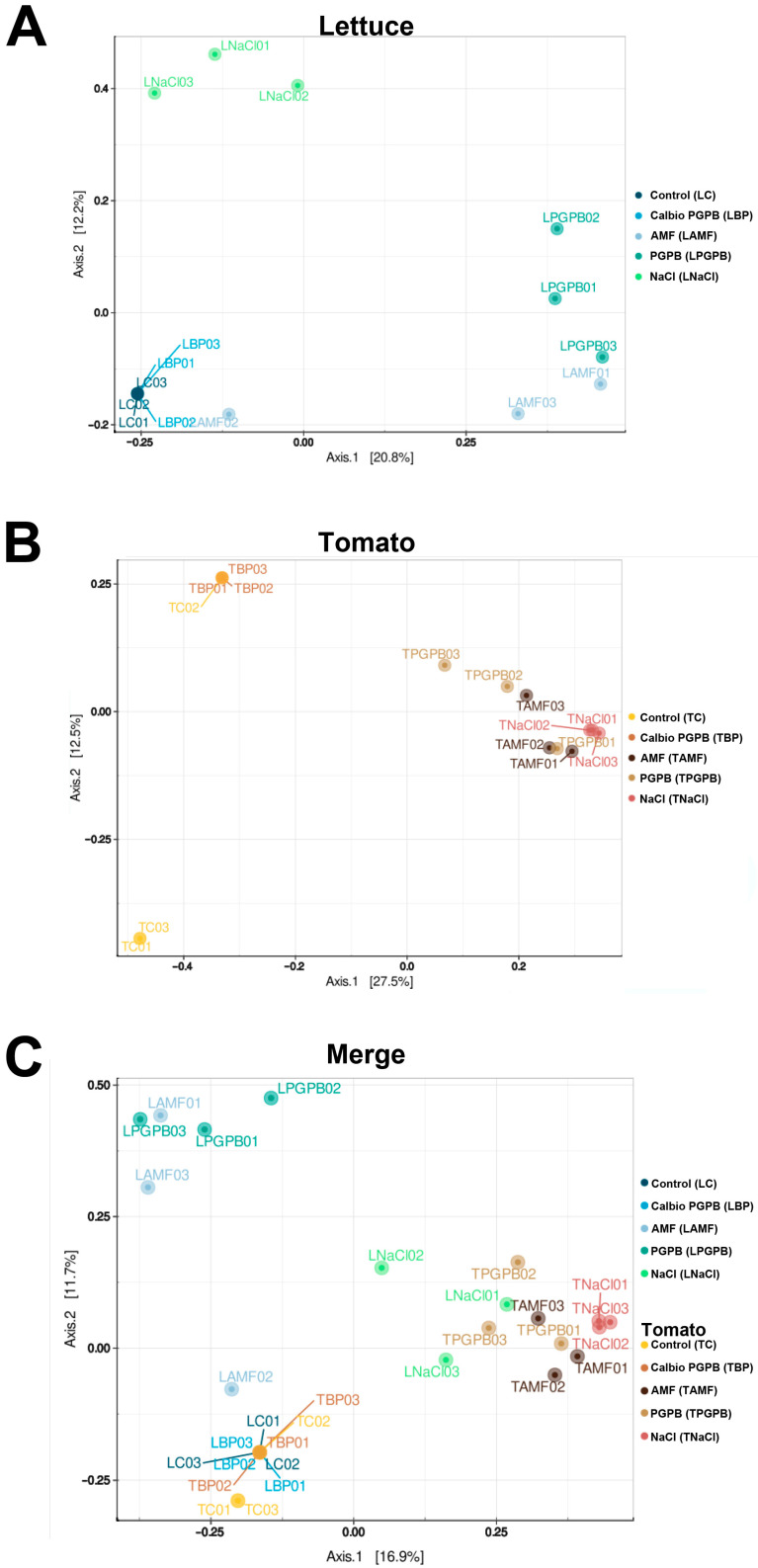
ASV-level β-diversity results of (**A**) lettuce samples, (**B**) tomato samples, and (**C**) both species merged. The treatments are control plants grown without any biostimulants (Control); plants treated with a combination of a non-microbial-based biostimulant, Calbio, and the microbial-based biostimulant BM08 (Calbio + PGPB); plants treated with a microbial-based biostimulant containing the arbuscular mycorrhizal fungi RI6E6 (AMF); and plants with the salt treatment (NaCl). Lettuce: Control (LC), Calbio + PGPB (LBP), AMF (LAMF), PGPB (LPGPB), and NaCl (NaCl). Tomato: Control (TC), Calbio + PGPB (TBP), AMF (TAMF), PGPB (TPGPB), and NaCl (TNaCl). Different biological replicates are numbered 01, 02, and 03.

The beta-diversity analysis at the ASV level (Figure 6) grouped replicates from the same treatment, suggesting that the treatment influenced the plants’ fungal community profile. In lettuce, similar to what we observed in bacteria, the control and the Calbio + PGPB samples were grouped, whereas PGPB and AMF were in another part of the graph, and NaCl defined an independent group (Figure 6A). In the case of tomato, the samples treated with NaCl, AMF, or PGPB were grouped, whereas the control samples were grouped with Calbio + PGPB (Figure 6B). When we merged the analysis from both species, we confirmed that, as observed for the bacterial communities, both controls grouped and the NaCl had a similar effect on the fungal profile in both plants. Additionally, in lettuce and tomato, the presence of the nonmicrobial biostimulant abolished the impact of the external application of PGPB, and it grouped with the control (Figure 6C).

## 4. Discussion

Several studies show that the endophytome is stable under different environmental conditions and that the plant selects its endophytome [42]. Studies like the one referenced are based on sampling the same plant in various natural environments. The data presented here challenge this perspective, at least in cultivated plants. We have performed a metataxonomic analysis to determine whether agronomical inputs (bacteria or fungi) or salt stress may affect the endophytome of crops and in which direction. Our results indicate that the addition of microbial biostimulants, a common practice in organic agriculture that is gaining popularity in conventional farming, significantly affects the composition of the endophytome. Furthermore, salt stress profoundly impacts this composition and increases its diversity. Previously, it has been reported that humic substances and osmotic stress may modulate the bacterial endomicrobiome of tomatoes [43]. The results of our systematic analysis show that this effect can be a complete change in the profile. In some cases we observed an increase in the presence of microbes that are known to be present in the human gut and usually associated with a healthy microbiome, but further research is required to confirm whether these changes may lead to healthier food.

The endophytome could be determinant not only for the nutritional properties of the food, but also for the plant’s health. Previous research has proven that the treatments used in this study enhance plant growth [16,17]. We can hypothesize that the changes in the endophytome could be, at least in part, responsible for the plants’ enhanced yield. In the lettuce samples, the presence of the potentially endophytic genus *Plectosphaerella* stood out, as well as that of another endophyte, *Cladosporium*. The genus *Pseudomonas* appeared in the control samples and several samples from the other groups, in some cases in significant abundance. This is a genus of interest since it has been seen as an endophyte in various crops, including tomatoes [44] and lettuce [45]. The increased presence has been shown to have beneficial effects, such as inhibiting pathogens or promoting growth. Therefore, the greater abundance of this genus has obvious agronomic and commercial interest. In addition, the genus *Pantoea*, which was detected in all the samples with NaCl and the PGPB group, also seems to confer advantages to the plant in which it is found, such as the promotion of growth and tolerance to stress, the assimilation of nitrogen and the control of diseases in several plants such as sugarcane [46]. *Stenotrophomonas*, present in all samples of these two groups, is also a genus that colonizes the endosphere of plants and the expression of certain functional traits that enhance the production of several metabolites and improve their survival in different habitats [47]. On the other hand, the relative abundance of *Sphingomonas* significantly increased in some treatments; it can also be beneficial for plants, promoting plant growth and tolerance to abiotic stresses [48]. It should be noted that no genus appeared to be significantly more present either in the AMF group or in the PGPB when compared to the NaCl group. Interestingly, we have observed that the combination of Calbio and PGPB confers enhanced growth in lettuce and tomato [16,17], but here we observe little effect on the microbiome, and the results are similar to the untreated (Control) group. We have seen that the stress response of these treated plants is attenuated, as the antioxidant response is much less than in the other treatments, so this combination appears to have a protective role. The interesting point is that in our previous research, the response of the Calbio + PGPB-treated plants under stress was similar to that of the control plants without stress. Here, this pattern is very similar at the endophytome level.

In tomato, the presence of *Enterobacter* in AMF-treated samples stood out (in abundances between 15 and 30%, compared to 0% in the rest of the tomato samples). This genus has already been linked to taxa associated with the application of AMF in some crops [49]. As for the group treated with PGPB, the dominant presence of *Pantoea* and, to a lesser extent, of another genus of the Erwiniaceae family stood out. *Pantoea*, as described above, is known for its potential to promote plant growth under different stress conditions, which is why it is often used as a biostimulant in other crops [50,51]. Massilia, another genus whose benefits for plant growth have been described [52], was also significantly more present in this group compared to control samples. On the other hand, other genera that can also benefit the plant, such as *Burkholderia* or *Mesorhizobium*, were not detected in practically any samples. The genus *Priestia*, used as an inoculum in the treated plants, does not appear in our analysis. Therefore, this genus cannot efficiently colonize other plant tissues beyond the root and rhizosphere.

We have also studied the presence of fungal phyla. Fungi represent less than 0,5% of gut microbiome, but the study of the fungi endophytome is of interest from the agronomic point of view, since some genera of fungi are responsible for significant economic losses in agricultural production. When studying the fungal composition of the endophytome, different genera were identified in the control samples of tomato compared to lettuce. Cladosporium stood out. This genus is endophytic in many plant species, including tomatoes, and has been shown to promote growth [53], as well as in lettuce [54] and has been routinely found in plant species grown in saline environments [41]. The genus *Plectosphaerella* predominated in lettuce, mainly in the PGPB group, although it was also seen in the AMF and NaCl groups. This genus was detected more or less systematically in a large part of the lettuce samples, so it could constitute a standard endophyte of this crop, as seen in other plants [39]. In addition, the genus *Talaromyces* was also detected in samples of the AMF, PGPB, and NaCl groups, both in lettuce and tomato. The potential endophytic character of this genus has already been described in recent studies [40].

The question we wanted to address is whether agronomical inputs or the cultivation conditions can modify the endophytome and increase the presence of microbes that are known to be beneficial for human health. Our study provides compelling evidence that applying microbial biostimulants and moderate salt stress can substantially enhance the microbial richness and composition of the endophytome in lettuce leaves and tomato fruits, both commonly consumed raw. The use of microbial biostimulants increased the presence of Firmicutes in lettuce. Firmicutes and Bacteroidetes are two main phyla found in the gut microbiome and their ratio is commonly used as an indicator of health status [55]. However, it is clear that more studies would be required to determine whether these beneficial organisms can actually have an impact on gut health through raw consumption of lettuce or tomatoes. Another outstanding question is the mechanism by which these microbial biostimulants may produce alterations in the composition of the microbes in the endophytome. This point will also require further study, but these observed effects could be due to the fact that the presence of AMF and PGPB in the soil alters the equilibrium in the rhizosphere. Although the two microorganisms used here are not present in the endophytome, their presence is known to alter the antioxidant and the hormonal response of the plant, and this could have an effect on the endophytome [16,17].

Another interesting outcome of the present study is the effect of salt stress. NaCl increases the Shannon and Simpson indices for bacteria and fungi. As discussed earlier, sodium is transported through the plants’ vascular system and accumulated in the aerial parts’ vacuoles and this accumulation has various effects on both the plant and the microbiome. Given the considerable variability in the salt tolerance of different microorganisms, this alters the equilibrium, affecting some of the most predominant taxa and genera, favoring some less represented taxa, and increasing biodiversity. It is known that irrigation with salty water diminishes yield but sometimes enhances the quality. In tomato, there are descriptions that moderate salt stress increases the flavor and the nutritional quality [56]. A mild salt stress increases sugar content in lettuce [57]. Our results indicate that the microbial biodiversity in the endophytome also increases. Importantly, salt stress—often viewed negatively in crop production—elicited the most consistent increase in microbial alpha-diversity across both crops, and significantly boosting the presence of *Firmicutes* and *Bacteroidota*, as mentioned above, two phyla frequently associated with a healthy human gut microbiota [58].

From a dietary perspective, these findings suggest that specific pre-harvest interventions—particularly microbial inoculation with PGPB and controlled exposure to salt stress—can modulate the edible plant microbiome. As fruits and vegetables are increasingly recognized not just as nutrient sources but also as microbial reservoirs, the presence of core gut-associated phyla and genera in the edible tissues underscores their functional relevance. Our data indicate that microbial biostimulants may alter the profile without reducing diversity. In most cases, we have confirmed that the treatments increased the presence of phyla usually considered as beneficial for gut microbiome and health status. However, more research using animal models and/or direct analysis of gut microbiota would be required to confirm whether the changes described in the present report lead to measurable effects on the nutritional quality of lettuce or tomatoes.

## 5. Conclusions

Most changes induced by the use of PGPB, AMF, or NaCl correlate with changes in the pattern of the endophytome. Specifically, microbial biostimulants increase the presence of *Firmicutes* in lettuce, and salt stress induces the accumulation of *Firmicutes* and *Bacteroidetes*. We have shown that using microbial biostimulants, which are very common in organic farming and gaining interest in conventional farming, or controlled stress could lead to vegetables with a different microbial profile and diversity. Further research should confirm whether these changes have a positive effect on gut health and a better diet.

## Data Availability

The original contributions presented in the study are included in the article/Supplementary Materials. Further inquiries can be directed to the corresponding author.

## Supplementary Materials

The following supporting information can be downloaded at: https://www.mdpi.com/article/10.3390/foods14193366/s1, Figure S1: Rarefaction curve obtained at the ASV level of the samples, once the chloroplastic and mitochondrial readings have been filtered. Figure S2: Rarefaction curve obtained at the ASV level of the samples, after filtering the readings from tomato and lettuce. Table S1: number of reads for each group.
