# Supplementary material for: Impact of Different Microbial Biostimulants and Salt Stress on the Endophytome of the Edible Part of Lettuce and Tomato Plants
Source: Foods. 2025 Sep 29;14(19):3366. doi: 10.3390/foods14193366 (PMC12523328; doi:10.3390/foods14193366)
Supplement: Supplementary file 1 [file foods-14-03366-s001.zip › Supplementary Figures S1 and S2.pptx]

## Slide 1
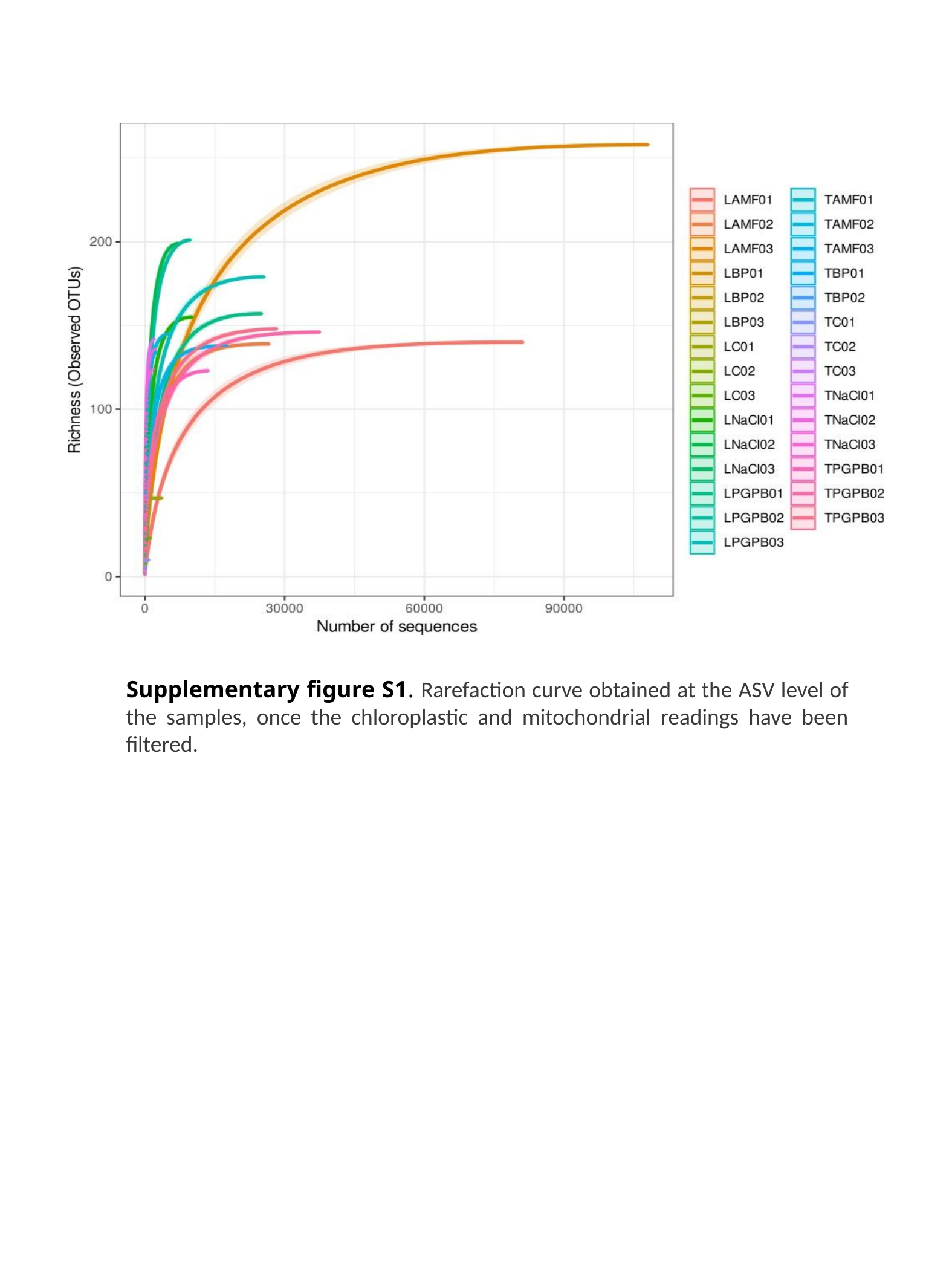

Supplementary figure S1. Rarefaction curve obtained at the ASV level of the samples, once the chloroplastic and mitochondrial readings have been filtered.

## Slide 2
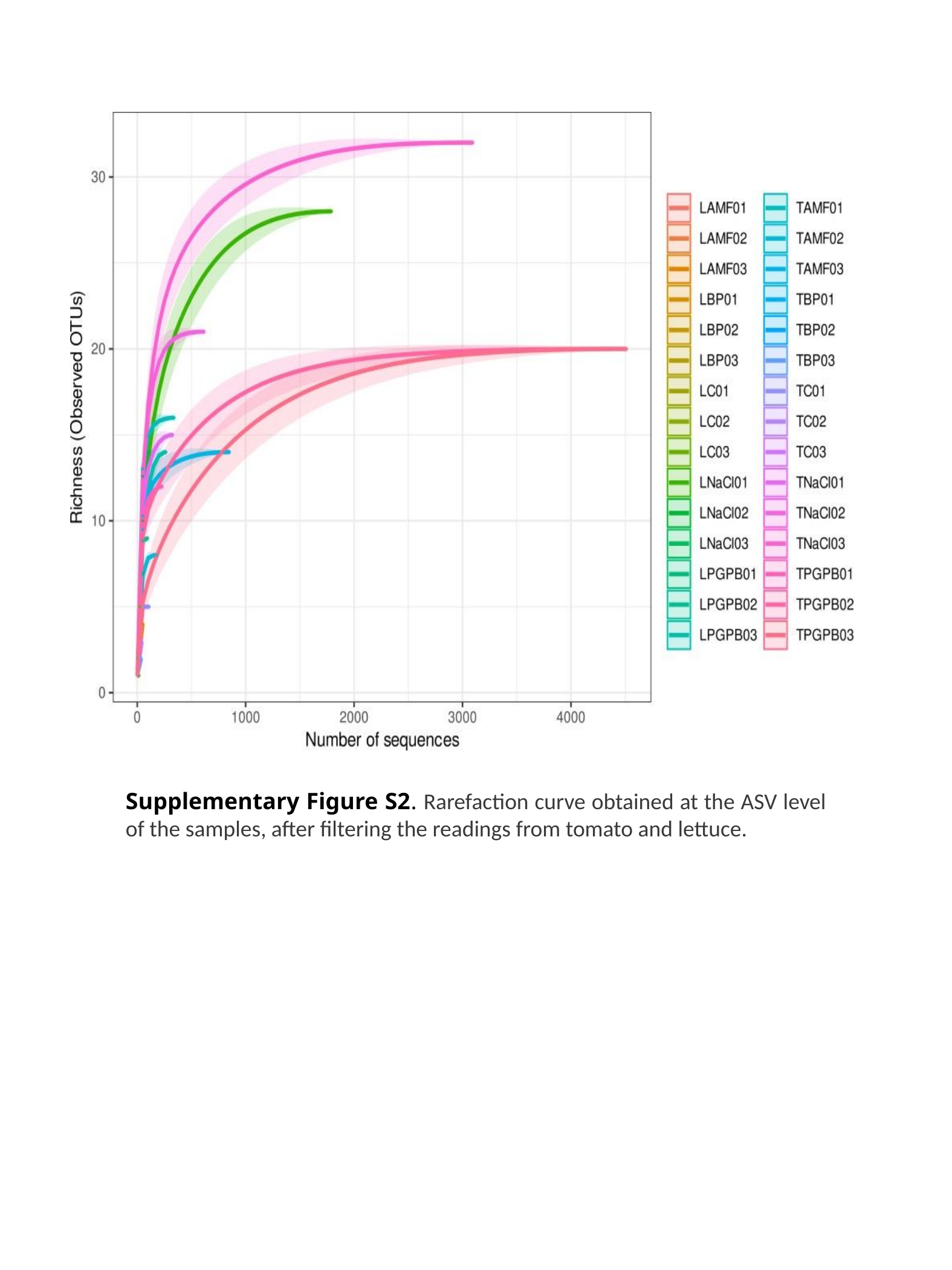

Supplementary Figure S2. Rarefaction curve obtained at the ASV level of the samples, after filtering the readings from tomato and lettuce.
